# Profile of an HIV Testing and Counseling Unit in Bangladesh: Majority of New Diagnoses among Returning Migrant Workers and Spouses

**DOI:** 10.1371/journal.pone.0141483

**Published:** 2015-10-29

**Authors:** Arunthia Zaidi Urmi, Daniel T. Leung, Vanessa Wilkinson, Mohammad Abdul Awal Miah, Mahfuza Rahman, Tasnim Azim

**Affiliations:** 1 Centre for HIV/AIDS, International Centre for Diarrheal Disease Research, Bangladesh, Dhaka, Bangladesh; 2 Hospitals, International Centre for Diarrheal Disease Research, Bangladesh, Dhaka, Bangladesh; 3 Centre for Vaccine Sciences, International Centre for Diarrheal Disease Research, Bangladesh, Dhaka, Bangladesh; 4 Institute for Breathing and Sleep, Austin Health, Heidelberg, Victoria, Australia; UCL Institute of Child Health, University College London, UNITED KINGDOM

## Abstract

**Introduction:**

Analysis of data from HIV testing and counseling (HTC) services provides an opportunity to identify important populations for targeting of HIV prevention efforts. Our primary aim was to describe the demographics of clients presenting to HTC in Bangladesh, a low HIV prevalence country. Our secondary aim was to determine the risk factors for HIV positivity among returning migrant workers who were tested.

**Methods:**

We performed a cross-sectional study of data collected between 2002 and 2010 from the first HTC service established in Bangladesh, located in three large cities.

**Results:**

8973 individuals attended HTC services, with 558 (6.2%) of clients testing positive for HIV, including 33 children. The majority of those who tested positive were aged 25–44 (71%), male (70%), and married (68%). Key populations considered at increased risk of HIV, such as female sex workers, people who inject drugs, and males who have sex with males accounted for only 11% of adults who tested positive. Notably, 75% of adults testing positive had a history of migrant work or was the spouse of a migrant worker. In multivariable logistic regression of those with a migrant work history presenting for HTC, we found rural residence, working in the Middle East, and longer duration of migrant work to be independently associated with testing positive, and female gender and higher level of education to be negatively associated.

**Conclusions:**

These data suggest that in Bangladesh, in addition to targeting traditional key populations, HIV prevention efforts should also focus on migrant workers and their spouses.

## Introduction

While marked reductions (>50%) of HIV incidence have been observed in 25 low- and middle-income countries in the past decade, Bangladesh is one of only nine countries in the world with an increase of >25% in estimated HIV incidence between 2001 and 2011 [1]. This is despite the fact that prevalence remains below 1%, even among those considered most at risk. While a concentrated HIV epidemic has been identified among people who inject drugs [2], other traditional key populations (KPs) at high risk, such as female sex workers and males who have sex with males, only account for a small percentage of People Living with HIV (PLHIV) in Bangladesh.[3] Although serological surveillances are in place for the aforementioned KPs, there is a paucity of data on the general population in Bangladesh due to its low HIV prevalence [4].

HIV testing and counseling (HTC), which includes Voluntary counselling and Testing (VCT) and Provider Initiated Testing and counselling (PITC), is an essential component of HIV prevention, care and support [5]. With the advent of treatment as prevention, HTC may act as the first entry point into the continuum of care, and at the same time provides an opportunity for education and counselling in a confidential environment. Monitoring of data from HTC sites can be used to effectively identify new populations at increased risk for HIV and to assess the impact of public health interventions [6].

Since the establishment of the first HTC centre in 2002 by the International Centre for Diarrheal Disease Research, Bangladesh (icddr,b), the number of HTC sites in Bangladesh has increased, and has also expanded across the country, through the efforts of various non-governmental organizations (NGOs). However, analysis characterizing utilization of HTC services in Bangladesh are lacking, and identifying the population groups testing positive for HIV at HTC sites could provide useful information for the optimization of prevention programs. Therefore, the primary aim of this study was to describe the demographics of clients presenting to HTC, with a focus on those who tested positive.

Labour migration is an increasing source of foreign exchange earnings for Bangladesh, and there are over 5 million Bangladeshis employed abroad [7]. We have previously shown that 77% of HIV-infected adults in our inpatient HIV ward have a history of external migration [8], and an estimated 50% of new HIV infections in 2010 in Bangladesh were among migrant workers [9]. However, there has not been a comprehensive description of HIV positive migrant workers, nor identification of risk factors for this population. Thus, a secondary aim of this study was to determine the risk factors for HIV positivity among returning migrant workers who present for testing.

## Materials and Methods

### Ethics statement

Written consent was taken from all clients attending the HTC services using a consent form stating that the client’s information can be used for future research and that confidentiality is respected for data provided. All patient records and information were anonymized and de-identified prior to analysis. Under the policy of the International Centre for Diarrheal Disease Research Ethical Review Committee, for this unlinked and anonymous analysis of service data, approval was not required.

### Study setting

We performed a cross-sectional study of data collected between 2002 and 2010 from the HTC units of icddr,b, known as “Jagori”, with one unit located in each of three major cities in Bangladesh. Icddr,b started the first HTC service in the country that provided a full range of counselling (pre-test, post-test and ongoing counselling) and testing (including confirmatory tests). The HTC Unit in Dhaka, the capital city (population 12 million), started in January 2002. At the end of 2003, in collaboration with Marie Stopes Clinic Society, two branches of the HTC unit of icddr,b, were opened: one in Chittagong (population 7.5 million), the second largest and a port city, and one in Sylhet (population 3.4 million), a city in the northeast. Information about services available was provided through word of mouth, and through NGOs and other organizations. The icddr,b HTC Unit has, from its onset, worked closely with Bangladeshi PLHIV support NGOs, which offer peer support and primary healthcare services, including distribution of antiretrovirals (ARVs).

### Data collection

The data used in this study were taken from anonymous HTC client records. A standardized recording format developed jointly by Jagori unit of icddr,b and Family Health International (fhi360), Bangladesh, adapted from the WHO [10], was used. The records, including anonymous questionnaire and pre-test and post-test counselling forms, were completed by HIV counsellors at the time of pre- and post-test counselling and entered into a computer using the Statistical Package for Social Sciences (Version 11.5 for windows, SPSS Inc., Chicago, IL, USA).

During pre-test counselling, the counsellors completed an anonymous questionnaire identifying the client only through a client registration number and laboratory number. The questionnaire is linked to the confidential detailed pre-test counselling form that is kept separately and used only by the counsellors for counselling. In addition to demographic information, the questionnaire collected information on client’s migrant work history. A “migrant worker” was defined as an individual who left Bangladesh to pursue employment (i.e. external migrant work). Details regarding internal migration were not collected.

### Data analysis

We compared demographic variables of migrant workers who tested positive versus those who tested negative, using Chi-squared test for categorical variables, and the Student t-test (for normally distributed data as assessed by Shapiro-Francia test) or Mann-Whitney U test for continuous variables. We used multivariable logistic regression to determine independent predictors of testing positive: we entered all variables into the regression model. For these comparisons, we used Stata version 13.1 (Stata Corp, College Station, TX). Statistical significance was defined as a two-tailed P value < 0.05. We generated graphs using GraphPad Prism 5.0 (GraphPad Software, Inc., La Jolla, CA).

## Results

**Fig 1:**
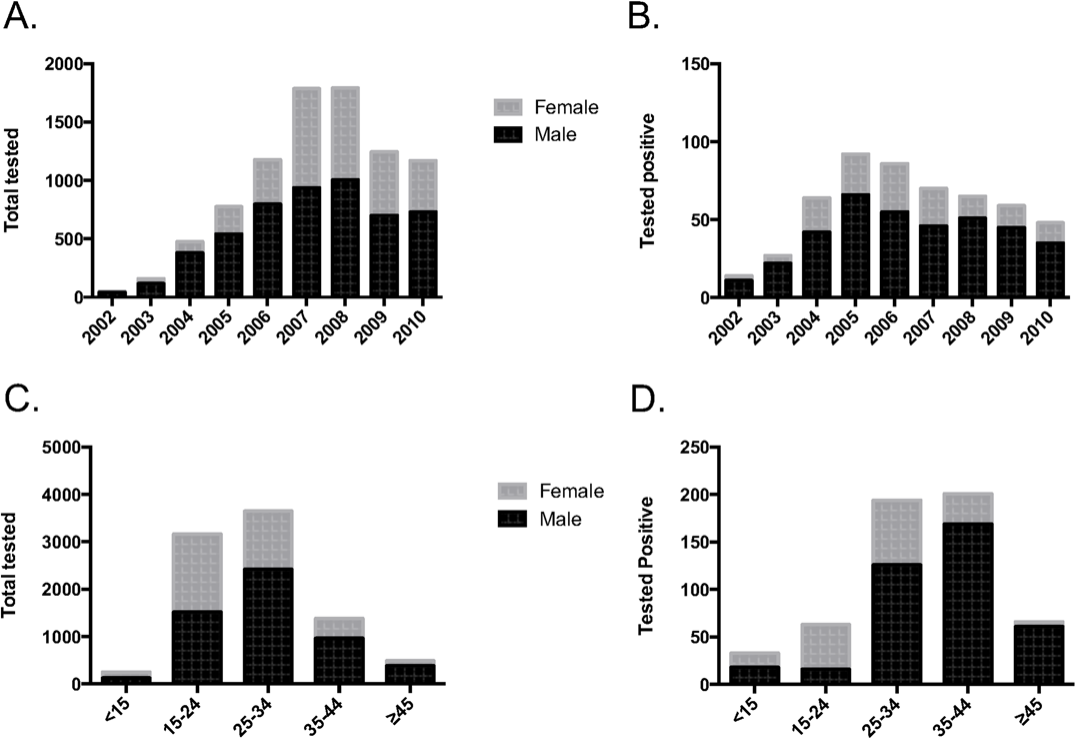
Number of clients presenting for VCT. A) All clients by year and sex, B) Those testing positive by year and sex, C) All clients by age group and sex, and D) Those testing positive by age group and sex.

**Fig 2:**
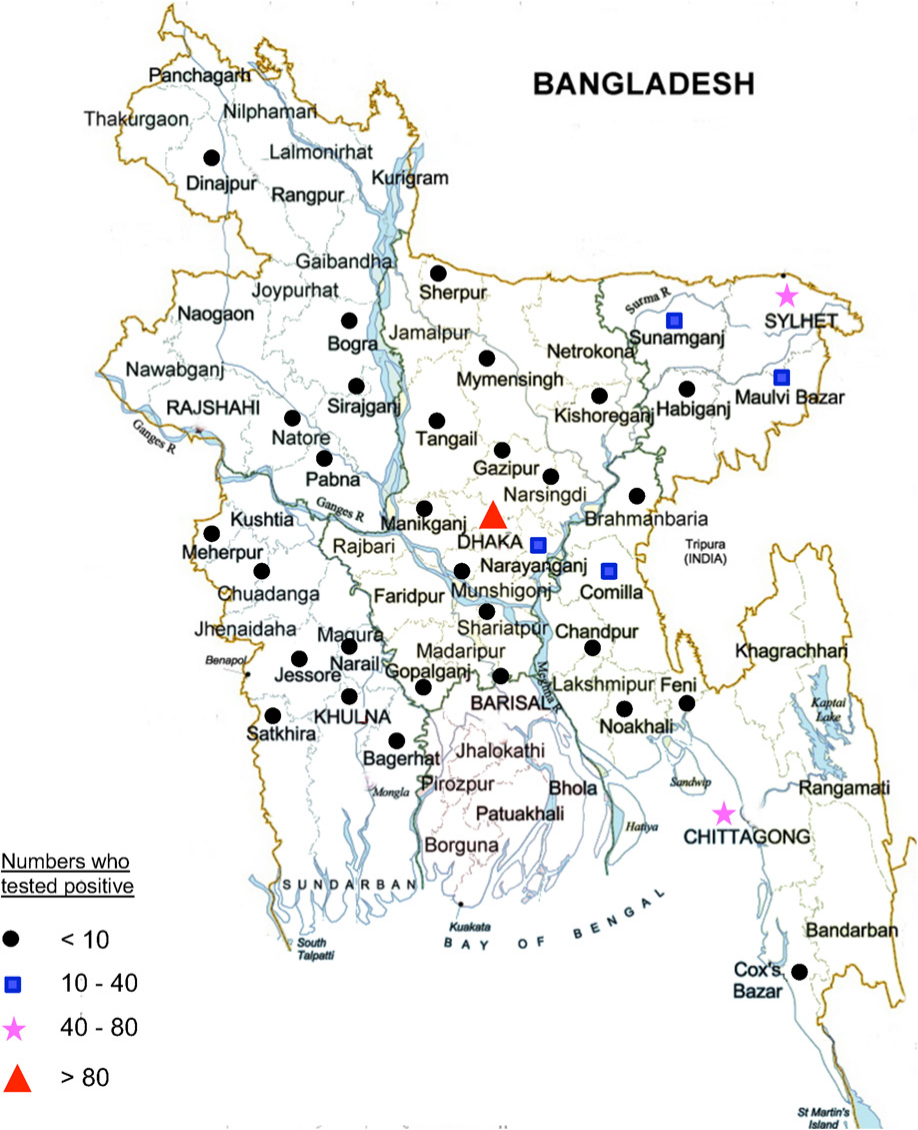
Current location, by district, of those who tested positive for HIV at the icddr,b HCT units.

A total of 8973 individuals attended for HTC at the Dhaka (n = 6456), Chittagong (n = 1621) and Sylhet (n = 896) sites between 2002 and 2010. Clients were mostly male (60.5% male, 39.2% female, 0.3% transgender) and had a mean age of 27.6 years (SD±8.9, range 0.7–76.0 years). The total number of clients attending HTC increased since the commencement of the service, with a peak of 1834 attendees in 2007 (Fig 1A). Overall, 6.2% (n = 558) of clients tested positive for HIV, of which 70% were male (Fig 1B). Of the small number of transgender clients (n = 27), only one tested HIV-positive. The proportion of clients testing HIV-positive progressively decreased during the study period, from a high of 24.6% in 2002, to a low of 3.8% in 2009. The 15–24 and 25–34 year age groups made up the majority of clients attending for HTC (35% and 41% of all clients, respectively, Fig 1C), while the 25–34 and 35–44 age groups most frequently tested positive for HIV (35% and 36% of all new diagnoses, respectively, Fig 1D). Of the 313 clients testing positive with home district data available, we found 36 of the 64 districts of Bangladesh represented (Fig 2).

Of the 558 clients who tested positive, 33 were children (<15 years old) and 525 adults. Of the children, 30 were the child of an HIV positive parent. Of the adults, 75% (395/525) were either a migrant worker (n = 309) or a non-migratory spouse of a migrant worker (n = 86). Traditionally recognized KPs such as female sex workers (n = 12), males who have sex with males (n = 11), and people who inject drugs (n = 36), accounted for 11% of all who tested positive for HIV. Over 95% (146 of 153) of adult females and 80% (296 of 372) of adult males who tested positive were married or formerly married.

### Migrant workers

**Fig 3:**
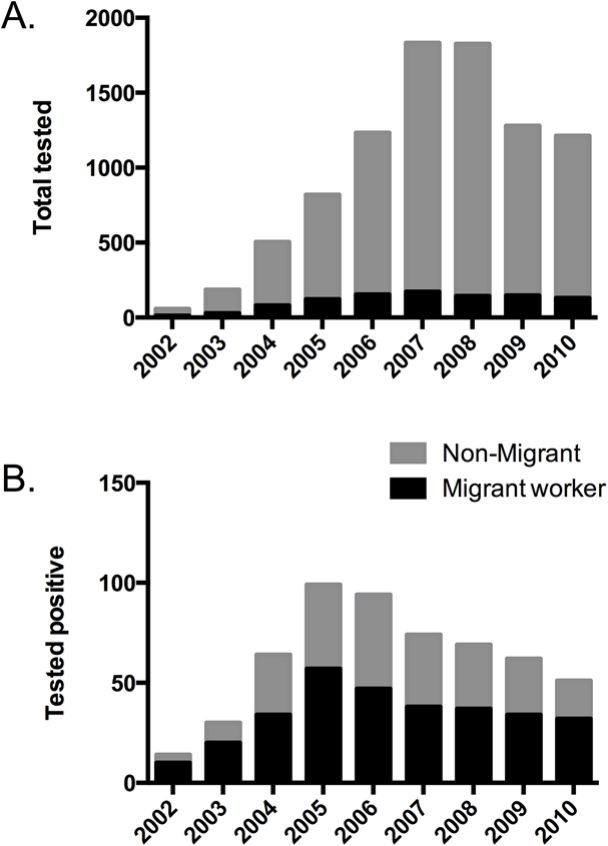
By year of study, number of migrants and non-migrants who: A) presented for testing, and B) tested positive.

We performed a sub-analysis on the population of migrant workers (n = 994) who presented for testing, of which 309 (31%) tested positive. Migrant workers accounted for between 50 to 70% of those who tested positive across all years examined (Fig 3). Table 1 shows the regions in which migrant workers who tested positive were employed. The Middle East was the most common region of migrant workers who came to HTC (51% of all migrants tested), the most common region of those who tested positive (71%), and migrants returning from there had the highest rate of positive tests (43%) compared to other regions. Including those who worked in multiple countries, the numbers were even higher when accounting for those who had ever worked in the Middle East.

**Table 1:** Migrant workers presenting to HTC, by geographic region of employment.

Region of employment	Total tested from region (n, %)	HIV positive (n, %)	% positive of those tested from region
Middle East	504 (51)	218 (71)	43
South Asia	177 (18)	29 (9)	16
Southeast Asia	125 (13)	36 (12)	29
Europe / North America	56 (6)	5 (2)	9
Multiple countries	72 (7)	12 (4)	17
Others*	49 (5)	7 (2)	14
Unknown	11 (1)	2 (1)	0.6
Total	994 (100)	309 (100)	31

* “Others” include Japan, Korea, Australia, and Libya.

We compared the demographic characteristics of migrant workers who tested negative (n = 685) versus those who tested positive (n = 309). Univariate analysis revealed that older age, rural residence, being married, ever working in the Middle East, and longer duration of migrant work were associated with testing positive, while female sex and having a higher graduate level of education were negatively associated (Table 2). In multivariable logistic regression (Table 3), we show that living in a rural area, having ever worked in the Middle East, and longer duration of migrant work were independent predictors of testing positive, while female gender and higher levels of education were negatively associated.

**Table 2:** Univariate comparison of demographic characteristics and behaviours of clients with history of migrant work presenting for HTC, by HIV test outcome.

Demographic variable	HIV negative (n = 685)	HIV positive (n = 309)	P-value*
Age, categories, n (%)			<0.001
15–24	83 (12)	15 (5)	
25–34	334 (49)	106 (34)	
35–44	195 (28)	146 (47)	
≥45	73 (11)	42 (14)	
Gender, female, n (%)	122 (18)	20 (6)	<0.001
Education, n (%)			<0.001
Illiterate	97 (14)	74 (24)	
Primary	110 (16)	83 (27)	
Secondary	202 (30)	114 (37)	
Higher secondary	76 (11)	27 (9)	
Graduate and above	198 (30)	10 (3)	
Marital status, Married, n (%)	399 (58)	244 (79)	<0.001
Current residence in Rural area, n (%)	140 (24)	170 (63)	<0.001
**Migration-related variable**			
Ever worked in Middle East, n (%)	323 (48)	228 (74)	<0.001
Duration of migrant work, n (%)			<0.001
≤2.0 years	155 (23)	16 (5)	
2.1–5.0 years	135 (20)	50 (16)	
5.1–10.0 years	91 (13)	57 (18)	
>10.0 years	60 (9)	89 (29)	
Unknown	244 (36)	97 (31)	
Spouse also migrant worker, n (%)	86 (13)	27 (9)	0.08

*P-values were computed using logistic regression.

**Table 3:** Independent predictors for testing positive among migrant workers presenting to HTC, by multivariable logistic regression analysis.

Variable	Odds Ratio	[95% CI]	P-value
Age category (years): 15–24	Ref		
25–34	0.72	0.34–1.51	0.38
35–44	0.93	0.42–2.05	0.85
≥45	0.53	0.21–1.33	0.18
**Gender, female**	**0.39**	**0.20–0.76**	**0.005**
Education: Illiterate	Ref		
Primary	0.72	0.43–1.23	0.23
Secondary	0.57	0.35–0.93	0.02
Higher secondary	0.50	0.26–0.96	0.04
**Graduate**	**0.10**	**0.05–0.24**	**<0.001**
**Rural residence**	**3.10**	**2.16–4.47**	**<0.001**
Married	1.24	0.80–1.91	0.33
Worked in Middle East	1.51	1.04–2.19	0.03
**Duration of migrant work:**			
**≤2.0 years**	Ref		
**2.1–5.0 years**	3.5	1.8–6.7	**<0.001**
**5.1–10.0 years**	5.1	2.6–10.1	**<0.001**
**>10.0 years**	12.2	6.0–24.8	**<0.001**
**Unknown**	3.3	1.7–6.4	**<0.001**
Spouse also migrant worker	1.18	0.62–2.22	0.62

We also compared the demographic characteristics of HIV positive migrants with HIV positive adult non-migrants and found that migrants were older (P < 0.001), less likely to be female (P < 0.001), lives in a rural area (P = 0.03), and married (P < 0.001). There were no differences between groups regarding the level of education (p = 0.18). In multivariable regression, living in a rural area (OR 2.5, CI 1.5–4.2, P = 0.001) and being married (OR 2.0, CI 1.1–3.6, P = 0.02) were independent positive predictors, and being a female (OR 0.04, CI 0.02–0.07, P < 0.001) was a negative predictor, of being an HIV-positive migrant compared to HIV-positive non-migrant.

## Discussion

Bangladesh is a low-income country with an estimated population of approximately 150 million persons and an HIV prevalence of less than 0.1% [1, 3]. Data on the prevalence and risk factors for HIV outside traditional KPs are limited. In this report, we describe the longitudinal experience of the first HTC unit in Bangladesh, and characterize risk factors for testing positive among migrant workers, who made up the majority of new HIV diagnoses.

From the establishment of HTC services in 2002 until 2010, we observed an overall increase in the utilization of HTC services at all three sites surveyed. Several factors likely played a role in this increase, including the initiation of rapid HIV tests with results given same day, initiation of free ARV distribution through self help NGOs for PLHIV, and increased referral from these and other NGOs. The decline of HTC utilization at icddr,b after 2007 was likely due to the establishment of HTC services by multiple other NGOs around that time period. We found that while those aged 15–24 and 25–34 years were most likely to utilize HTC services, the 25–34 and 35–44 years age groups made up the majority of those who tested positive. These findings are consistent with an Indian study showing the 35–44 years age group to have the highest odds of testing positive [11]. We postulate that the high positivity rate in older age groups seen in our study is likely a reflection of delayed HIV testing and diagnosis [12], though recent acquisition of those in this age group cannot be ruled out. This finding underscores the need for educational efforts aimed at the middle aged and older population, with the goal of early testing and diagnosis for those at risk for HIV.

We found that the proportion of clients testing positive progressively decreased during the study period, from nearly 25% at the service’s inception in 2002 to less than 5% in 2010. This is a reflection of the increase in number of clients presenting for testing, and is due to a better understanding and awareness of HIV/AIDS amongst both the general public and local healthcare providers. While public health awareness campaigns implemented by both governmental and non-governmental organizations through the past decade have likely contributed to this increase in testing, further work is required to improve disease detection and in the prevention of transmission.

Labour migration plays a large role in the global economy, and remittances from migrant labourers can provide substantial income for families in low-income countries. The International Organization for Migration estimates that over 5 million Bangladeshis are employed abroad [7], with nearly $14 billion in foreign remittances in 2013 alone, approaching that of the top export industry, that of ready-made garments [13]. We have shown that, despite being only a small percentage (11%) of HTC clients, individuals associated with a history of migrant work accounted for the majority (75%) of HIV-positive diagnoses. Moreover, increasing duration of migrant work was independently associated with testing positive. This is consistent with data from the National AIDS and STD Program of the Govt. of Bangladesh, showing that approximately 50% of new HIV infections in 2010 were among migrant workers [9]. Migrants and mobile populations are at high risk of HIV infection all over the world, and a systematic review had reported that HIV prevalence among migrants is higher compared to the general population [14]. We have previously reported that rural Bangladeshi men who lived apart from their spouse were more likely to have had sex with a female sex worker or with another male [15]. A qualitative study of Bangladeshi returning migrant workers revealed that female workers are victims of sexual exploitation by both male co-workers and employers [16]. In that study, 80% of male migrant workers reported sexual intercourse, of whom only 5% used condoms. Similarly, a study of male migrant workers in India, over half of whom were married, showed that 31% reported sex with a paid or unpaid non-spousal partner in previous 2 years [17]. Furthermore, in a case control study from rural India, the prevalence of HIV was significantly higher among males with a migration history, and significantly higher among women with migrant husbands than those without [18]. In our study, 79% of returning migrants who tested positive were married, and 15% of those who tested positive were non-migrant spouses of migrant workers, who likely acquired their infection from their migrant spouse. Aside from transmission to spouse, the contribution of returning migrant workers to overall transmission in the general population is unclear.

We found that over 70% of migrants who tested positive had worked in the Middle East, and history of working there was an independent predictor for testing positive. Our group has recently used phylogenetic analysis of virus from Bangladeshi HIV patients to show at least seven strains that clustered with African strains, and appear to have been introduced by returning migrants from the Middle East [19]. Recent events, including the deaths of migrant workers involved in the construction of new football stadiums, have afforded increasing attention on the healthcare issues of the migrant worker population. A recent study revealed that 71% of new HIV diagnoses in Saudi Arabia were found in expatriates [20]. Given that millions of migrant workers from South Asian countries are working in the Middle East, including over 560,000 new workers from Bangladesh alone in 2011, further study of this population is needed to identify appropriate strategies for prevention of HIV acquisition abroad.

Among migrant workers who presented for testing, residence in a rural area was found to be an independent predictor of testing positive. Furthermore, examination of district of residence data revealed that while the largest concentrations of individuals who tested positive resided in districts with large cities, many districts without large cities were also represented. A recent population-based cohort study from India revealed a two-fold higher HIV incidence among rural than urban adults [21]. Given that educating those living in rural areas may require strategies different than that for urban dwellers, any HIV prevention effort targeting migrant workers must take into account the high numbers of workers originating from rural areas.

Additionally, and as a consequence of the above, vertical transmission of HIV continues to play a prominent role in the increasing prevalence of the disease in Bangladesh. In our study, we identified 33 children to be HIV positive, at least 30 of which were children of parents who had suspected or confirmed HIV infection. Earlier work from our institution has shown that HIV-infected children attending our inpatient ward were diagnosed at a median of 5.2 years after birth [22], likely a result of a lack of detection of infected mothers. In that study, 75% of children had parents with a migration history, underscoring the contribution of migrant work to paediatric HIV infections in this country. Appropriate prenatal counselling and availability of HTC during perinatal health visits, along with implementation of a systematic Prevention of Mother to Child Transmission (PMTCT) program, have proven to be effective in averting transmission of HIV to children.

This study has several notable limitations. Given that we focused only on those presenting for HTC, the largest limitation of this study is the selection and referral biases inherent to institution-based studies. Thus, the risk factors identified for HIV positivity can only be applied to those who seek or are referred for testing services. Despite this, risk factors identified may serve as a proxy of risk factors for disease among the general population, especially in the absence of a comprehensive national sero-surveillance in this group. A second limitation of this study is that self-reporting of risk factors by clients may lead to a reporting bias, and thus rates of MSM, people who inject drugs, and sex workers may be underestimated. Thirdly, as this was a retrospective analysis using client forms completed over several years, the analysis was limited to variables that had been uniformly entered and complete.

In conclusion, we describe the implementation and uptake of a multi-site HTC program. We have demonstrated that the majority of new diagnoses of HIV in Bangladesh occur in returning migrant workers and their spouses. Our data suggest that promoting HIV awareness among migrant workers may add to the success of HIV prevention efforts in the country.
